# The joint effect of gestational diabetes mellitus and hypertension contribute to higher risk of diabetes mellitus after delivery: a nationwide population-based study

**DOI:** 10.1186/s12884-023-05829-6

**Published:** 2023-07-26

**Authors:** Ho-Poh Kek, Yu-Tsun Su, Shu-Jiin Tey, Ming-Chun Yang, Li-Ching Chang, Yun-Hsiang Hung, Ching-Chung Tsai

**Affiliations:** 1 Department of Pediatrics, E-Da Hospital, I-Shou University, No.1, Yi-Da Road, Yan-Chao District, Kaohsiung City, 82445 Taiwan, R.O.C.; 2 School of Medicine for International Students, College of Medicine, I-Shou University, No. 8, Yi-Da Road, Yan-Chao District, Kaohsiung City, 82445 Taiwan, R.O.C.; 3 Department of Obstetrics and Gynecology, E-Da Hospital, I-Shou University, No.1, Yi-Da Road, Yan-Chao District, Kaohsiung City, 82445 Taiwan, R.O.C.

**Keywords:** Gestational diabetes mellitus, Pregnancy-induced hypertension, Diabetes mellitus, Hypertension, Metabolic syndrome

## Abstract

**Background:**

Gestational diabetes mellitus (GDM) and pregnancy-induced hypertension (PIH) are known risk factors for postpartum diabetes mellitus (DM) and hypertension, respectively. This study aimed to examine the association between the co-occurrence of GDM and PIH and the subsequent development of diabetes mellitus (DM), hypertension, and metabolic syndrome.

**Methods:**

A cohort study was conducted using data from the Taiwan National Health Insurance Research Database (TNHIRD). The study population included 2,297,613 pregnant women with no history of certain medical conditions who gave birth between 2004 and 2015. The women were classified into four cohorts based on their medical history: GDM cohort, PIH cohort, both GDM and PIH cohort, and normal cohort (without GDM and PIH).

**Results:**

The GDM cohort had a higher risk of developing DM, hypertension, and metabolic syndrome than the normal cohort, with hazard ratios of 7.07, 1.54, and 2.51, respectively. The PIH cohort also had an increased risk for these conditions compared with the normal cohort, with hazard ratios of 3.41, 7.26, and 2.68, respectively. The cohort with both GDM and PIH had the highest risk of developing postpartum DM, hypertension, and metabolic syndrome, with hazard ratios of 21.47, 8.02, and 5.04, respectively, compared with the normal cohort.

**Conclusion:**

The cohort of patients with both GDM and PIH had the highest impact on developing postpartum DM compared with either condition alone cohort. Furthermore, the co-occurrence of both conditions increases the risk, with a higher likelihood of developing postpartum DM than hypertension or metabolic syndrome.

## Background

Gestational diabetes mellitus (GDM) is a form of diabetes that manifests during pregnancy; it is characterized by high blood glucose levels that were absent or well-controlled before pregnancy. On the other hand, pregnancy-induced hypertension (PIH), also known as gestational hypertension, refers to the development of high blood pressure after 20 weeks of gestation in previously normotensive women [1, 2]. Both GDM and PIH can have significant implications for the health and well-being of both the mother and the developing fetus. In Taiwan, the prevalence of diabetes mellitus (DM), hypertension, and metabolic syndrome among pregnant women is reported to be 8.8%, 25%, and 12.0%, respectively [3–5]. Therefore, these issues require considerable research attention.

GDM is a condition characterized by impaired glucose tolerance, insulin resistance, and dysfunction of pancreatic β-cells [6]. It can result in fetal macrosomia, hyperinsulinemia, and an increased risk of childbirth complications [7]. Furthermore, women with GDM are also at a higher risk of developing type 2 DM and cardiovascular/cerebrovascular diseases postpartum [8, 9]. A study has shown that 15–60% of women with GDM develop type 2 DM within 5–15 years of pregnancy [10]. Additionally, women with GDM are diagnosed with type 2 DM approximately 7.7 years earlier (95% confidence interval [CI]: 5.8–9.6) than those without pregnancy complications. GDM is associated with a threefold higher risk (hazard ratio [HR]: 3.68, 95% CI: 2.77–4.90) of type 2 DM later in life [11]. A systematic review revealed that women with a history of GDM have a significantly elevated risk of developing type 2 DM (weighted relative risk [RR]: 13.2) and cardiovascular disease (weighted RR: 2.0) compared with those without GDM [12]. Another study demonstrated that women with GDM have a two-fold increased risk of future cardiovascular events, independent of concurrent type 2 DM. These risks manifest within the first decade of pregnancy [9].

PIH is recognized worldwide as a significant cause of maternal and perinatal mortality [13]. Moreover, hypertension during pregnancy may lead to long-term metabolic and vascular abnormalities, thereby increasing the risk of cardiovascular disease [14]. Studies have established that PIH increases the risk of chronic hypertension (RR: 2.3–11), subsequent development of type 2 DM (RR: 1.8), and cardiovascular death (RR: 2.1) [15]. Another study indicated that PIH increased the risk of subsequent hypertension by 5.31 times and the risk of developing type 2 DM by 3.12 times [16]. Additionally, PIH has been found to significantly increase the likelihood of various health conditions, including ischemic heart disease (HR: 1.44), myocardial infarction (HR: 1.75), myocardial infarct death (HR: 3.00), heart failure (HR: 1.78), ischemic stroke (HR: 1.59), kidney disease (HR: 1.91), and diabetes mellitus (HR: 1.52) [17]. Furthermore, a study discovered that women who experienced hypertensive disorders of pregnancy (HDP) had elevated blood pressure levels 2.5 years after giving birth. Another study showed that 1513 women faced a heightened risk of hypertension five years after delivery, even after accounting for confounding factors (odds ratio [OR]: 7.1) [18, 19].

Few studies have explored the relationship between the coexistence of GDM and PIH and the risk of developing type 2 DM, hypertension, or metabolic syndrome after pregnancy, despite the individual risks [20]. Therefore, the primary aim of this study was to examine the impact of GDM and PIH on the development of these conditions following childbirth, with a specific focus on the combined effects of GDM and PIH. The objective of this study was to provide healthcare providers with valuable insights for developing effective postpartum follow-up plans and facilitating early detection and treatment of these conditions.

## Materials and methods

### Data source

As per regulations in Taiwan, birth attendants are required to report relevant information within seven days of birth, which is recorded in the Birth Certificate Application linked to the Taiwan National Health Insurance Research Database (TNHIRD). This application includes important fields such as maternal background (nationality and residence), pregnancy details (risk factors and procedures), newborn information (weight and anomalies), and paternal information (nationality and registration). In this study, the diagnoses of GDM and PIH were obtained from pregnancy-related details recorded in Birth Certificate Applications between 2004 and 2015. In Taiwan, GDM is detected through an oral glucose tolerance test after the 20th week of pregnancy, whereas PIH is diagnosed by monitoring blood pressure readings of at least 140/90 mmHg, taken at least four hours apart [1, 2].

In addition, the data used in this study were obtained from the TNHIRD, which covers over 99% of the medical reimbursement claims made under the Taiwan NHI program. The database used in this study includes all outpatient and inpatient medication records. TNHIRD encrypts patient identity data to protect patient privacy and security. Diagnosis and drug coding were based on the International Classification of Diseases, Ninth or Tenth Revision, Clinical Modification (ICD-9-CM or ICD-10-CM, respectively). This study received approval from the Institutional Review Board of the E-DA Hospital (EMRP-108–061). Informed consent was waived by the Institutional Review Board of the E-DA Hospital as the study only utilized encrypted data from TNHIRD and did not involve direct contact with the study participants. All methods were conducted in adherence to applicable guidelines.

### Subject selection

To enhance the homogeneity of the study population and minimize confounding factors, patients with pre-existing illnesses such as type 1 DM, type 2 DM, essential hypertension, secondary hypertension, hyperlipidemia, severe psychosis, mood disorders, metabolic syndrome, any severe illness certified in the registry before pregnancy, toxemia of pregnancy, and missing data were excluded from the study. This exclusion strategy enabled a more accurate assessment of the association between GDM, PIH, and the outcomes of interest. The study population was categorized into the GDM cohort, PIH cohort, both GDM and PIH cohort, and the normal cohort (without GDM and PIH) according to the Birth Certificate Application. Mothers from the GDM cohort, PIH cohort, both GDM and PIH cohort were matched individually with mothers from the normal cohort at a ratio of 1:4, based on age, index day, and gestational age. The end of the follow-up period was defined as the day of diagnosis of type 2 DM, hypertension, metabolic syndrome, or death, or 31 December 2017. This variation in the follow-up period was accounted for in the study design to ensure a minimum of two years of follow-up data for all included mothers.

### Statistical analysis

The statistical analysis for this study initially involved analyzing the baseline characteristics and birth records of all the cohorts. The cohorts with GDM, PIH, and both GDM and PIH were compared with their corresponding normal cohorts to assess the varying risks of developing type 2 DM, hypertension, and metabolic syndrome. To evaluate these risks, a stratified Cox regression hazard model was used, which was adjusted for maternal age, nationality, mode of delivery, gestational week, and residential area. All data management and HR calculations were performed using SAS software for Windows (version 9.4; SAS Institute, Cary, NC, USA).

## Results

**Fig.1:**
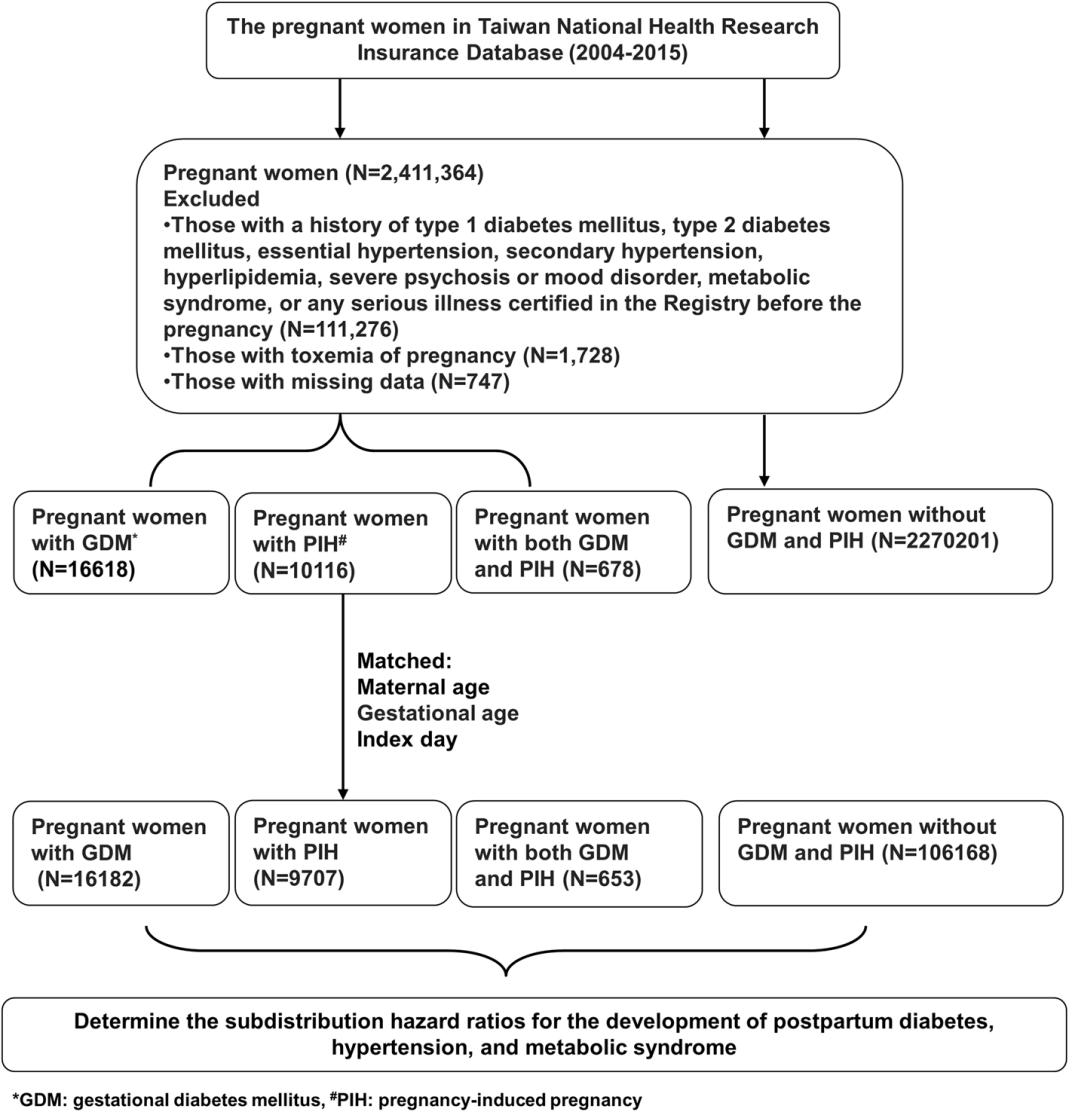
Flow chart of the study design based on data from the Taiwan National Health Insurance Research Database

In this study we identified 2,297,613 pregnant women without a history of type 1 or 2 DM, essential hypertension, secondary hypertension, hyperlipidemia, severe psychosis or mood disorder, metabolic syndrome, serious illness certified in the registry prior to pregnancy, or toxemia during pregnancy, and no missing data. Of these women, 16,618 were diagnosed with GDM, 10,116 with PIH, 678 with both GDM and PIH, and 2,270,201 had no history of GDM or PIH (control cohort). To ensure comparability, the three cohorts were individually matched with the control cohort at a ratio of 1:4, based on age, gestational age, and index day (Fig. 1).

After matching, the study included 16,182 women with GDM and 64,728 healthy controls. Comparisons of baseline characteristics between the GDM and control cohorts showed no significant differences in age, gestational age, nationality, and mode of delivery. However, the GDM cohort had a significantly higher likelihood of developing DM (6.40% vs. 0.99%, *P* < 0.05), hypertension (2.99% vs. 2.12%, *P* < 0.05), and metabolic syndrome (6.57% vs. 2.76%, *P* < 0.05) than the control cohort. For the PIH cohort, there were 9,707 matched cases and 38,828 controls. Comparisons of baseline characteristics between the PIH and control cohorts showed no significant differences in age or gestational age. However, the PIH cohort had a significantly higher likelihood of developing DM (3.99% vs. 1.27%, *P* < 0.05), hypertension (18.69% vs. 2.82%, *P* < 0.05), and metabolic syndrome (8.76% vs. 3.63%, *P* < 0.05) than did the control cohort. Finally, the study included 653 women with both GDM and PIH and 2,612 control. Baseline characteristic comparisons between the both GDM and PIH cohort and the control cohort showed no significant differences in age, infant sex, gestational age, and nationality. However, the cohort with both GDM and PIH had a higher likelihood of developing DM (16.85% vs. 0.96%, *P* < 0.05), hypertension (18.99% vs. 2.87%, *P* < 0.05), and metabolic syndrome (14.09% vs. 3.37%, *P* < 0.05) than the control cohort (Table 1).

**Table 1:** Baseline characteristics of the study cohorts

Event	GDM study		*p*-value	PIH study		*p*-value	GDM&PIH study			*p*-value
	Control cohort *N* = 64,728	GDM cohort *N* = 16,182		Control cohort *N* = 38,828	PIH cohort *N* = 9707		Control cohort *N* = 2612	GDM&PIH cohort *N* = 653		
Age of mother	33.44 ± 4.20	33.44 ± 4.19	> 0.05	31.42 ± 4.87	31.43 ± 4.87	> 0.05	33.66 ± 4.62		33.69 ± 4.62	> 0.05
Infant Gender			< 0.05			< 0.001				> 0.05
Girl	30,927 (47.78)	7490 (46.29)		18,068 (46.53)	4792 (49.37)		1233 (47.21)		317 (48.55)	
Boy	33,801 (52.22)	8692 (53.71)		20,760 (53.47)	4915 (50.63)		1379 (52.79)		336 (51.45)	
Gestational age	38.01 ± 1.80	38.01 ± 1.80	> 0.05	37.27 ± 2.40	37.27 ± 2.40	> 0.05	37.36 ± 2.11		37.36 ± 2.11	> 0.05
Birth weight	3035.93 ± 479.07	3140.39 ± 535.74	< 0.01	2897.89 ± 566.69	2767.9 ± 677.39	< 0.001	2910.52 ± 530.40		3062.75 ± 702.55	< 0.001
Nationality	51,961 (80.28)	13,015 (80.43)	> 0.05	27,236 (70.15)	7064 (72.77)	< 0.001	2059 (78.83)		529 (81.01)	> 0.05
Mode of delivery			> 0.05			< 0.001				< 0.001
NSD	38,357 (59.26)	9673 (59.78)		22,472 (57.88)	3936 (40.55)		1448 (55.44)		291 (44.56)	
CS	26,371 (40.74)	6509 (40.22)		16,356 (42.12)	5771 (59.45)		1164 (44.56)		362 (55.44)	
Residential area			< 0.001			< 0.001				< 0.001
Northern Taiwan	21,918 (33.86)	7115 (43.97)		11,898 (30.64)	2706 (27.88)		893 (34.19)		231 (35.38)	
Northern-Central Taiwan	11,607 (17.93)	3420 (21.13)		7132 (18.37)	1565 (16.12)		416 (15.93)		140 (21.44)	
Central Taiwan	12,310 (19.02)	2024 (12.51)		7877 (20.29)	2054 (21.16)		508 (19.45)		94 (14.40)	
Southern-Central Taiwan	7657 (11.83)	1402 (8.66)		4958 (12.77)	1371 (14.12)		321 (12.29)		74 (11.33)	
Southern Taiwan	9307 (14.38)	1654 (10.22)		5599 (14.42)	1316 (13.56)		389 (14.89)		68 (10.41)	
Eastern Taiwan	1929 (2.98)	567 (3.50)		1364 (3.51)	695 (7.16)		85 (3.25)		46 (7.04)	
Develop diabetes mellitus	639 (0.99)	1035 (6.40)	< 0.001	494 (1.27)	387 (3.99)	< 0.001	25 (0.96)		110 (16.85)	< 0.001
Develop hypertension	1372 (2.12)	484 (2.99)	< 0.001	1096 (2.82)	1814 (18.69)	< 0.001	75 (2.87)		124 (18.99)	< 0.001
Develop metabolic syndrome	1787 (2.76)	1063 (6.57)	< 0.001	1411 (3.63)	850 (8.76)	< 0.001	88 (3.37)		92 (14.09)	< 0.001

*GDM* Gestational diabetes mellitus, *PIH* Pregnancy-induced hypertension, *NSD* Normal spontaneous delivery, *CS* Caesarean section

A stratified Cox regression hazards model analysis was conducted to determine whether GDM or PIH was the primary risk factor for developing postpartum DM, hypertension, and metabolic syndrome. The HR of the GDM cohort developing DM was 7.07 (95% CI:6.40–7.82, *P* < 0.05); the HR of developing hypertension was 1.54 (95% CI:1.39–1.71, *P* < 0.05); and the HR of developing metabolic syndrome was 2.51 (95% CI:2.32–2.71, *P* < 0.05). Compared with caesarean section, vaginal delivery had a significantly lower HR for developing DM, hypertension, and metabolic syndrome. Additionally, older mothers had a significantly higher HR for developing DM, hypertension, or metabolic syndrome (Table 2).

**Table 2:** Prediction for occurrence of postpartum diabetes, hypertension and metabolic syndrome in gestational diabetes mellitus cohort

Event	Develop DM	*p*-value	Develop HTN	*p*-value	Develop MS	*p*-value
	Adjusted HRs		Adjusted HRs		Adjusted HRs	
GDM vs. Control cohort	7.07 (6.40–7.82)	< 0.001	1.54 (1.39–1.71)	< 0.001	2.51 (2.32–2.71)	< 0.001
Age of mother	1.04 (1.02–1.05)	< 0.001	1.09 (1.08–1.11)	< 0.001	1.06 (1.05–1.07)	< 0.001
Nationality	0.99 (0.89–1.10)	> 0.05	0.90 (0.81–0.99)	< 0.05	0.98 (0.90–1.06)	> 0.05
NSD vs. CS	0.76 (0.69–0.84)	< 0.001	0.68 (0.62–0.75)	< 0.001	0.75 (0.70–0.81)	< 0.001
Gestational age	0.90 (0.88–0.92)	< 0.001	0.89 (0.88–0.91)	< 0.001	0.94 (0.92–0.96)	< 0.0001
Residential area						
Northern Taiwan	0.54 (0.43–0.68)	< 0.001	0.60 (0.47–0.76)	< 0.001	0.74 (0.61–0.89)	< 0.05
Northern-Central Taiwan	0.51 (0.40–0.64)	< 0.001	0.69 (0.54–0.88)	< 0.05	0.66 (0.54–0.81)	< 0.001
Central Taiwan	0.64 (0.50–0.82)	< 0.05	0.73 (0.57–0.94)	< 0.05	0.63 (0.51–0.77)	< 0.001
Southern-Central Taiwan	0.62 (0.48–0.81)	< 0.05	0.70 (0.54–0.91)	< 0.05	0.81 (0.65–0.99)	< 0.05
Southern Taiwan	0.63 (0.49–0.80)	< 0.05	0.78 (0.60–1.00)	< 0.05	0.70 (0.57–0.87)	< 0.05
Eastern Taiwan	REF.		REF.		REF.	

*HR* Hazard ratio, *DM* Diabetes mellitus, *HTN* Hypertension, *MS* Metabolic syndrome, *GDM* Gestational diabetes mellitus, *NSD* Normal spontaneous delivery, *CS* Caesarean section, *REF.* Reference

For the PIH cohort, the HR of developing DM was 3.41 (95% CI:2.98–3.91, *P* < 0.05); the HR of developing hypertension was 7.26 (95% CI:6.73–7.84, *P* < 0.05); and the HR of developing metabolic syndrome was 2.68 (95% CI:2.46–2.93, *P* < 0.05), compared with the control cohort. Similar to the GDM cohort, the PIH cohort had a significantly lower risk of developing DM, hypertension, and metabolic syndrome with vaginal delivery than with caesarean section. Moreover, older mothers were at a significantly higher risk of developing these conditions (Table 3).

**Table 3:** Prediction for occurrence of postpartum diabetes, hypertension and metabolic syndrome in pregnancy-induced hypertension cohort

Event	Develop DM	*p*-value	Develop HTN	*p*-value	Develop MS	*p*-value
	Adjusted HRs		Adjusted HRs		Adjusted HRs	
PIH vs. Control cohort	3.41 (2.98–3.91)	< 0.0001	7.26 (6.73–7.84)	< 0.0001	2.68 (2.46–2.93)	< 0.0001
Age of mother	1.05 (1.04–1.07)	< 0.0001	1.06 (1.06–1.07)	< 0.0001	1.06 (1.05–1.07)	< 0.0001
Nationality	0.94 (0.82–1.08)	> 0.05	0.92 (0.85–0.99)	> 0.05	1.00 (0.91–1.09)	> 0.05
NSD vs. CS	0.64 (0.56–0.74)	< 0.0001	0.78 (0.72–0.84)	< 0.0001	0.75 (0.69–0.82)	< 0.0001
Gestational age	0.93 (0.91–0.95)	< 0.0001	0.94 (0.93–0.95)	< 0.0001	0.95 (0.93–0.96)	< 0.0001
Residential area						
Northern Taiwan	0.69 (0.51–0.93)	< 0.05	0.66 (0.56–0.77)	< 0.0001	0.85 (0.70–1.03)	> 0.05
Northern-Central Taiwan	0.66 (0.48–0.92)	< 0.05	0.75 (0.63–0.89)	< 0.05	0.75 (0.61–0.92)	< 0.05
Central Taiwan	0.81 (0.59–1.10)	> 0.05	0.76 (0.65–0.89)	< 0.05	0.75 (0.61–0.91)	< 0.05
Southern-Central Taiwan	0.77 (0.56–1.07)	> 0.05	0.71 (0.60–0.85)	< 0.05	0.78 (0.63–0.96)	< 0.05
Southern Taiwan	0.83 (0.60–1.14)	> 0.05	0.86 (0.73–1.02)	> 0.05	0.78 (0.63–0.96)	< 0.05
Eastern Taiwan	REF.		REF.		REF.	

*HR* Hazard ratio, *DM* Diabetes mellitus, *HTN* Hypertension, *MS* Metabolic syndrome, *PIH* Pregnancy-induced hypertension, *NSD* Normal spontaneous delivery, *CS* Caesarean section, *REF.* Reference

In both GDM and PIH cohort, the HRs for developing DM, hypertension, and metabolic syndrome compared with the control cohort were 21.47 (95% CI:13.8–33.4, *P* < 0.05), 8.02 (95% CI:5.99–10.74,* P* < 0.05), and 5.04 (95% CI:3.74–6.80, *P* < 0.05), respectively. Taken together, these results indicate that women in both GDM and PIH cohorts had the highest likelihood of developing DM, hypertension, and metabolic syndrome after pregnancy, as shown in Table 4.

**Table 4:** Prediction for occurrence of postpartum diabetes, hypertension and metabolic syndrome in both gestational diabetes mellitus and pregnancy-induced hypertension cohort

Event	Develop DM	*p*-value	Develop HTN	*p*-value	Develop MS	*p*-value
	Adjusted HRs		Adjusted HRs		Adjusted HRs	
GDM&PIH vs. Control cohort	21.5 (13.8–33.4)	< 0.0001	8.02 (5.99–10.7)	< 0.0001	5.04 (3.74–6.80)	< 0.0001
Age of mother	0.99 (0.96–1.03)	> 0.05	1.05 (1.02–1.08)	< 0.05	1.04 (1.01–1.07)	< 0.05
Nationality	0.73 (0.50–1.06)	> 0.05	0.73 (0.54–0.99)	< 0.05	0.76 (0.55–1.04)	> 0.05
NSD vs. CS	0.68 (0.48–0.98)	< 0.05	0.82 (0.61–1.09)	> 0.05	0.77 (0.57–1.04)	> 0.05
Gestational age	0.91 (0.86–0.98)	< 0.05	0.93 (0.88–0.99)	< 0.05	0.95 (0.89–1.01)	> 0.05
Residential area						
Northern Taiwan	0.80 (0.42–1.51)	> 0.05	0.61 (0.36–1.03)	> 0.05	0.76 (0.42–1.36)	> 0.05
Northern-Central Taiwan	0.70 (0.36–1.39)	> 0.05	0.50 (0.28–0.90)	< 0.05	0.68 (0.36–1.27)	> 0.05
Central Taiwan	0.57 (0.27–1.19)	> 0.05	0.41 (0.22–0.76)	< 0.05	0.46 (0.23–0.90)	< 0.05
Southern-Central Taiwan	0.73 (0.35–1.50)	> 0.05	0.65 (0.36–1.18)	> 0.05	0.81 (0.42–1.55)	> 0.05
Southern Taiwan	0.83 (0.40–1.72)	> 0.05	0.73 (0.41–1.32)	> 0.05	0.45 (0.22–0.93)	< 0.05
Eastern Taiwan	REF.		REF.		REF.	

*HR* Hazard ratio, *DM* Diabetes mellitus, *HTN* Hypertension, *MS* Metabolic syndrome, *GDM* Gestational diabetes mellitus, *PIH* Pregnancy-induced hypertension, *NSD* Normal spontaneous delivery, *CS* Caesarean section, *REF.* Reference

## Discussion

This study demonstrated that pregnant women with GDM face a higher risk of developing type 2 DM (adjusted HR: 7.07, 95% CI: 6.40–7.82) than that of hypertension and metabolic syndrome after pregnancy when compared to the control cohort. These results are consistent with those of a previous Danish study that reported that women with GDM had a six-fold higher risk of developing DM than those without GDM (RR: 6, 95% CI: 4–11), with a particularly high incidence rate observed 15 years after pregnancy and beyond the age of 40 [21]. The risk of developing metabolic syndrome and subsequently DM is nearly tripled in GDM women after 6 years of pregnancy or after the age of 40 when compared with non-diabetic women [22]. Furthermore, a systematic review and meta-analysis confirmed that women with GDM have a substantially elevated risk of developing type 2 DM (RR: 7.43, 95% CI: 4.79–11.51) compared to women with normal blood glucose levels during pregnancy [23]. Another systematic review demonstrated that women who experienced GDM were significantly more likely to develop type 2 DM compared to those without a history of GDM (weighted RR: 13.2, 95% CI: 8.5–20.7) [12]. Similarly, a Netherlands study indicated that women with a history of GDM face a 4.0-fold increased risk of developing type 2 DM compared to those without GDM (HR: 3.68, 95% CI: 2.77–4.90) [11]. The collective evidence from these studies, including this one, consistently highlights a significant association between GDM and the subsequent development of type 2 DM in women. Furthermore, our study also identified an association between pregnant women with GDM and the subsequent risk of hypertension and metabolic syndrome, albeit with lower adjusted HRs than those for type 2 DM. Similar findings have been reported in Asian studies, which identified GDM as a significant independent risk factor for both DM and metabolic syndrome [24–26].

This study found that pregnant women with PIH have a significantly higher susceptibility to developing hypertension (adjusted HR: 7.26, 95% CI: 6.73–7.84) than the occurrence of type 2 DM and metabolic syndrome after pregnancy compared to the control cohort. These findings are consistent with those of previous studies, including an investigation that reported a significant increase in the risk of hypertension for the cohort with HDP/non-GDM, relative to the reference group (adjusted HR: 11.2, 95% CI: 8.19–15.2). Similarly, the HDP/non-GDM cohort demonstrated an elevated risk of developing type 2 DM compared to the comparison cohort (adjusted HR: 3.15, 95% CI: 2.55–3.89) [20]. Additionally, a Danish study provided evidence supporting the association between gestational hypertension and subsequent hypertension risk (5.31-fold increased risk, range: 4.90 to 5.75), as well as the risk of subsequent type 2 DM (3.12-fold increased risk, range: 2.63 to 3.70) [16]. Furthermore, a retrospective cohort study conducted in Taiwan reported that the incidence rate of DM after PIH was five times higher than that in non-PIH women [27], which is consistent with our study's 3.4-fold HR. A review article emphasized the increased risk of chronic hypertension (2.3 to 11-fold) and subsequent type 2 DM (1.8-fold) associated with PIH [15]. These findings align with those of our research, highlighting the substantial risk of developing hypertension and type 2 DM following gestational hypertension. Notably, the risk of developing hypertension was higher than developing type 2 DM (adjusted HRs: 7.2 versus 3.14). Furthermore, our study identified an association between pregnant women with PIH and the subsequent risk of metabolic syndrome, albeit with a slightly lower risk than that of type 2 DM. In addition, a Korean study revealed that PIH increases the risk of developing metabolic syndrome in the future with a shorter time to development than in the general population [28]. Therefore, our study adds to the existing body of evidence on the association among PIH, hypertension, type 2 DM, and metabolic syndrome in pregnant women.

Few studies have investigated the combined effects of GDM and PIH on the development of type 2 DM, hypertension, and metabolic syndrome. One study found that women with both HDP and GDM have a significantly higher risk of developing type 2 DM than those with hypertension, although both risks are elevated compared to women without these complications. In the general population, the incidence of type 2 DM is higher than that of hypertension. The adjusted HRs for subsequent type 2 DM and hypertension were similar among women with both HDP and GDM, with values up to 16.8 (95% CI: 11.8–24.1) and 16.2 (95% CI: 13.2–19.9), respectively [20]. Moreover, younger women may experience a more pronounced combined effect of HDP and GDM on the risk of postpartum incident diabetes mellitus, which may be attributed to underlying metabolic and vascular conditions rather than specific pregnancy complications [20]. In contrast to the previous studies, our research demonstrated that maternal age had minimal impact on the future risk of postpartum incident diabetes. Additionally, in our study, pregnant women with both GDM and PIH had a significantly higher likelihood of developing type 2 DM after pregnancy (adjusted HR: 21.5, 95% CI: 13.8–33.4) than the risks of developing hypertension and metabolic syndrome. Furthermore, our study showed that the risk of developing post-pregnancy DM is significantly higher in women with both GDM and PIH than in those with only GDM (adjusted HRs: 21.5 versus 7.07), whereas the risk of developing post-pregnancy hypertension was only slightly higher in women with both GDM and PIH than in those with PIH only (adjusted HRs:8.02 versus 7.26). Hence, our findings enhance the understanding of the combined effects of GDM and PIH on health outcomes, emphasizing the importance of considering both conditions in clinical management and follow-up care.

In this study, a lower likelihood of developing DM, hypertension, or metabolic syndrome after pregnancy was observed in women who underwent vaginal delivery than in those who underwent caesarean section. This difference may be attributed to obstetricians’ tendency to choose caesarean sections for high-risk cases. Additionally, the results showed that delivery at a later gestational age was associated with a decreased risk of developing DM, hypertension, or metabolic syndrome after pregnancy. This observation may be attributed to the fact that individuals with severe PIH or GDM often require early delivery, which increases their vulnerability to developing type 2 DM and hypertension during follow-up period [11, 29].

Nationality did not appear to have a significant influence on the development of DM, hypertension, or metabolic syndrome after pregnancy in this study, likely because the majority of the participants were Taiwanese. However, geographical location was found to affect the likelihood of developing these conditions, with women residing in eastern Taiwan exhibiting higher HRs. This may be attributed to the limited availability of medical and educational resources, as well as the comparatively poorer socioeconomic environment in this mountainous and remote region.

The use of the Birth Certificate Application to identify pregnant women with GDM and PIH, along with a large sample size from the TNHIRD, enhanced the study’s robust methodology and generalizability. This study contributes significantly to the existing literature by highlighting the fact that GDM and PIH are significant risk factors for postpartum type 2 DM, hypertension, and metabolic syndrome. Importantly, this study revealed a higher risk of developing type 2 DM in pregnant women with both GDM and PIH than in those with only one of these condition.

## Limitations

This study has some limitations. First, it did not include information on personal factors, such as dietary habits, lifestyle, education level, body weight, and socioeconomic status of pregnant women. Second, the study lacked laboratory data and information on compliance with GDM and PIH treatment during pregnancy, which could affect the development of DM, hypertension, and metabolic syndrome later in life [30, 31]. Third, pregnant women who were included closer to 2015 had a limited follow-up period of only two years. Finally, a family history of DM, hypertension, or metabolic syndrome among pregnant women was not considered in this study.

## Conclusion

This study highlights GDM and PIH as significant risk factors for postpartum DM, hypertension, and metabolic syndrome. Pregnant women with both GDM and PIH are at a higher risk of developing DM than either condition cohort. Furthermore, the risk is further elevated in those with both conditions, with DM being more likely than hypertension or metabolic syndromes. Hence, maintaining healthy habits during pregnancy can help prevent GDM and PIH. Adequate postpartum care is suggested for women with both conditions to minimize the risk of DM.

## Data Availability

The data related to the study can be provided upon a reasonable request. Please contact Dr. Ching-Chung Tsai (email: u101130@gmail.com) to obtain the requested data.

## Abbreviations

CI: Confidence interval
DM: Diabetes mellitus
GDM: Gestational diabetes mellitus
HDP: Hypertensive disorders of pregnancy
HR: Hazard ratio
OR: Odds ratio
PIH: Pregnancy-induced hypertension
RR: Relative risk
TNHIRD: Taiwan National Health Insurance Research Database
